# Pan-Cancer Analysis Confirms the Prognostic and Immunological Implications of the 1,25-Dihydroxy Vitamin D3 Receptor in Cervical Squamous Cell Carcinoma

**DOI:** 10.7759/cureus.66743

**Published:** 2024-08-12

**Authors:** Israa Faris M Faris, Noon Ibrahim, Tomador S Zeanelabdeen, Mohamed Alfaki

**Affiliations:** 1 Faculty of Veterinary Medicine, University of Khartoum, Khartoum, SDN; 2 Department of Fertilization and Artificial Insemination, Istanbul University-Cerrahpasa, Istanbul, TUR; 3 Department of Public Health/Health Policy, Planning and Management, Ahfad University for Women, Omdurman, SDN; 4 Department of Allergy and Immunology, Private Practice, Mecca, SAU; 5 Department of Research, Sidra Medicine, Doha, QAT

**Keywords:** immune cell infiltration, cervical squamous cell carcinoma, vitamin d3 receptor, volcano plot, prognostic biomarkers, vdr, cesc

## Abstract

Vitamin D receptor (VDR), specifically the 1,25-dihydroxy form, holds significant importance in various types of cancer, including cervical squamous cell carcinoma (CESC), which poses a significant public health challenge. A pan-cancer analysis was conducted on VDR in CESC, with a focus on its expression and relationship with immune infiltration and genetic alterations.

Bioinformatics databases, including TIMER, GEPIA, UALCAN, cBioportal, and Kaplan-Meier Plotter, have been utilized. VDR expression in CESC has been validated using publicly available data. Results were significantly upregulated (P=0.05) in THCA, BRCA, KICH, LUAD, LIHC, STAD, UCEC, CESC, CHOL, ESCA, and HNSC samples.

We analyzed the correlation between VDR expression and various clinicopathological factors such as age, race, and cancer stage. VDR expression was significantly upregulated across all age groups, with the highest levels observed in older adults followed by young and middle-aged adults. VDR gene expression was significantly elevated across all races, including Caucasians, African-Americans, and Asians, compared to that in the normal group. Furthermore, VDR expression was significantly upregulated in cancer stages 1, 2, 3, and 4, with the highest increase observed in stage 3 compared to that in normal individuals.

We analyzed the expression of the VDR in relation to immune cell type and tumor cell purity in CESC. Our results indicated that VDR expression was positively correlated with neutrophils and dendritic cells and negatively correlated with tumor cell purity in CESC patients. There was no significant correlation between VDR expression and the abundance of B cells, CD8+ T cells, CD4+ T cells, and macrophages.

Our study found no significant effect of VDR expression on patient prognosis, although it was positively correlated with CD4+ T cells. The Cox proportional hazards model indicated that age and immune cells did not significantly affect prognosis. Most VDR mutations are concentrated in diffuse large B-cell lymphoma, with an amplification frequency of 4% and a deep deletion frequency of 2.2%. GEO confirmed VDR expression in CESC, identifying 1515 upregulated and 1877 downregulated genes, with volcano plots showing CESC downregulation in patients.

## Introduction

Cervical squamous cell carcinoma (CESC) is a prevalent type of cancer that affects the cervix and is the second leading cause of cancer-related death in women worldwide [1,2]. Vitamin D receptor (VDR) has a significant impact on the immune system. It has been shown to modulate the activity of key immune cells such as T cells, B cells, and macrophages. Previous studies have suggested that VDR may play a role in the development and progression of different cancers; however, more research is needed to fully comprehend its implications [3]. In cutaneous squamous cell carcinoma, VDR may influence the tumor microenvironment and the immune response against cancer cells [4,5].

In endometrial cancer, displacement of VDR has been associated with a lower histological grade, indicating a potential prognostic role, which suggests that VDR may function as a useful biomarker to predict prognosis and guide treatment decisions in CESC [4]. Therefore, understanding the prognostic and immunological implications of the VDR in CESC is crucial because it could potentially lead to the development of novel treatments and therapies for this type of cancer [5].

Research is scarce on the prognostic and immunological implications of VDR in CESC, particularly, using bioinformatic tools. In this study, we aimed to fill this knowledge gap by conducting a pan-cancer analysis of VDR in CESC using bioinformatics tools to better understand its prognostic and immunological implications [6]. The objective of this study was to conduct a pan-cancer analysis of VDR in CESC using bioinformatic tools to better understand its prognostic and immunological implications. We hypothesized that VDR plays a significant role in the prognosis and immune response of patients with CESC.

## Materials and methods

Transcriptional expression analysis of genes

TIMER Tumor Immune Estimation Resource (TIMER 2.0)

The TIMER database provides computational biologists with the ability to analyze the association between gene expression and the abundance of immune infiltrates in various cancers [7]. First, we employed TIMER (<https://cistrome.shinyapps.io/timer/>) to investigate the differential expression of the tumor vitamin D (1,25-dihydroxy vitamin D3) receptor across all pan-cancer analyses. We then displayed the distribution of the VDR gene expression levels using box plots.

Gene Expression Profiling Interactive Analysis (GEPIA)

GEPIA is a web-based tool that supplies exhaustive and interactive analyses of gene expression data from The Cancer Genome Atlas (TCGA) and Genotype-Tissue Expression (GTEx) projects (2015 A global reference for human genetic variation) [8]. The GEPIA database was used to assess the expression of the VDR gene in diverse cancers to ascertain whether there were notable differences in the expression profile. The "expression analysis BoxPlot module" was utilized to investigate VDR expression in tumors and normal tissues, with the following criteria being applied: P= 0.05, and log2|fold change| (FC) ≥ 1.

University of Alabama at the Birmingham Cancer Data Analysis (UALCAN) Portal

The UALCAN database is an instinctive web platform for cancer data analysis. It offers access to public datasets, such as The Cancer Genome Atlas, facilitating precise examination of gene expression, correlations, and patient survival rates. Additionally, it allows comparisons of tumor samples based on clinicopathological characteristics, such as cancer stage, age, sex, and race [9, 10].In this study, the UALCAN database was used to investigate the expression of VDR in CESC, which was subsequently validated using the GEPIA and TIMER databases. Additionally, we used the UALCAN database to explore the potential associations between VDR and clinicopathological factors, including age, gender, and race.

Kaplan-Meier Plotter

The Kaplan-Meier plotter (https://kmplot.com/analysis/) is a tool that can evaluate the relationship between the expression levels of all genes (mRNA, miRNA, protein, and DNA) and patient survival. In this study, we used the Kaplan-Meier plotter to assess the survival rates to relation VDR and CESC.

 *cBioPortal*

The cBioPortal (https://www.cbioportal.org/) for Cancer Genomics is an open-source platform that facilitates the exploration, visualization, and analysis of multidimensional cancer genomics data, allowing researchers to quickly and easily investigate complex genomic alterations across samples, patients, and studies, including gene-level data analysis, network visualization, survival analysis, and examination of genetic alterations in individual tumor samples [11,12]. In this study, we used the cBioPortal platform to assess genetic modifications present in BMX across a range of cancer types. 

Gene Expression Omnibus Validation of VDR Expression Using the Volcano plot

We utilized publicly available datasets from the National Center for Biotechnology Information (NCBI) database. Our differential expression analysis was performed using the GEO2R tool (<https://www.ncbi.nlm.nih.gov/geo/geo2r>), which is a resource provided to help users query and download experiments and curated gene expression profiles [13]. This enabled us to identify the significance of VDR in cervical squamous cell carcinoma by using the GSE122697 dataset. To visualize the results, we used the www.bioinformatic.com.cn database for the visualization of data, to generate a volcano plot. Statistical analyses were conducted based on the criteria of |Log2FC| > 1 and adj p-value < 0.05 to identify differentially expressed genes.

## Results

Expression of VDR across various cancers

**Figure 1:**
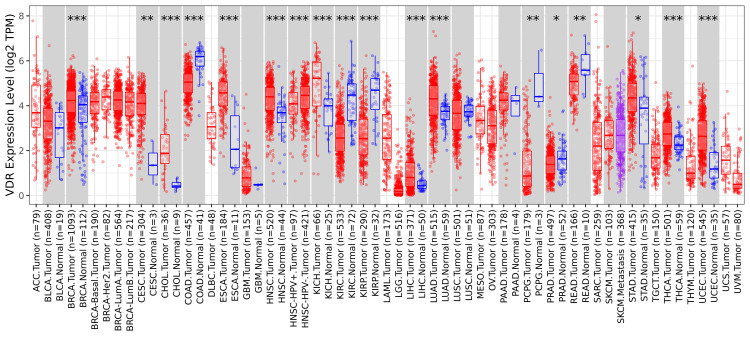
VDR expression using the TIMER database VDR: 1,25-dihydroxy vitamin D3 receptor; CESC: cervical squamous cell carcinoma; num(T): number of tumor samples, num(N): number of normal samples. P value: * p<0.05, **p<0.01, ***p<0.00

In this study, the expression of VDR was investigated in numerous cancers and normal tissue samples using TIMER 2.0. The outcomes showed substantial downregulation or upregulation of the gene in tumors relative to normal tissues for each cancer type, as illustrated by the gray columns when normal data were available (*p< 0.05; **p<0.01; ***p<0.001). The VDR expression was down-regulated in seven types of cancer: COAD (colon adenocarcinoma), KIRP (kidney renal papillary cell carcinoma), KIRC (renal clear cell carcinoma), READ (rectal adenocarcinoma), PCPG (Pheochromocytoma and Paraganglioma), and PRAD (prostate adenocarcinoma); in contrast, VDR expression was upregulated in cancer samples of THCA (thyroid carcinoma), BRCA (breast invasive carcinoma), KICH (kidney chromophobe), LUAD (lung adenocarcinoma), LIHC (liver hepatocellular carcinoma), STAD (stomach adenocarcinoma), UCEC (uterine corpus endometrial carcinoma), CESC (cervical squamous cell carcinoma), CHOL (cholangiocarcinoma), ESCA (esophageal carcinoma), and HNSC (head and neck squamous cell carcinoma) (Figure 1).

**Figure 2:**
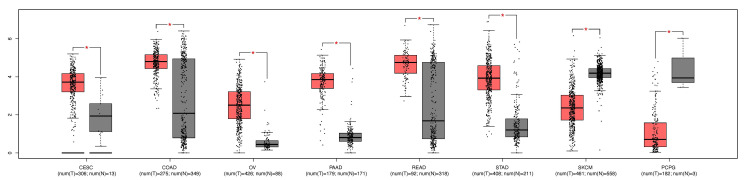
Expression of VDR in several tumors and CESC patients using the GEPIA database Pan-cancer analysis conducted to evaluate the expression of the VDR gene by using GEPIA. VDR: 1,25-dihydroxy vitamin D3 receptor; CESC: cervical squamous cell carcinoma; num(T): number of tumor samples, num(N): number of normal samples. P value: * p<0.05, **p<0.01, ***p<0.001

Moreover, we explored the differential expression of VDR in CESC by using the GEPIA database; the results showed that the gene (p-value < 0.05) significantly was upregulated (Figure 2).

**Figure 3:**
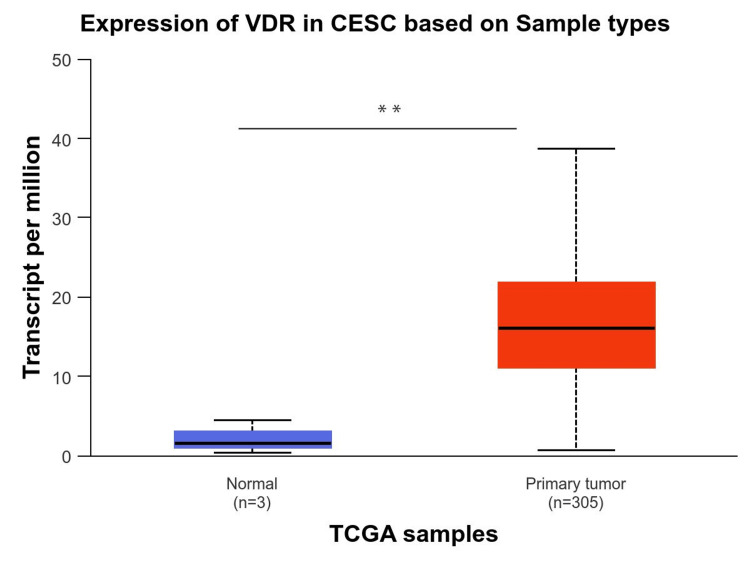
Expression of VDR in CSEC patients using the UALCAN database VDR: 1,25-dihydroxy vitamin D3 receptor; CESC: cervical squamous cell carcinoma; num(T): number of tumor samples, num(N): number of normal samples. P-value: * p<0.05, **p<0.01, ***p<0.001

In addition, we used the UALCAN database to analyze VDR expression in CESC (Figure 3). The results indicated that this gene was significantly upregulated in the tumor tissue (p-value < 0.05).

Clinical parameter analysis of the VDR gene across CESC

**Figure 4:**
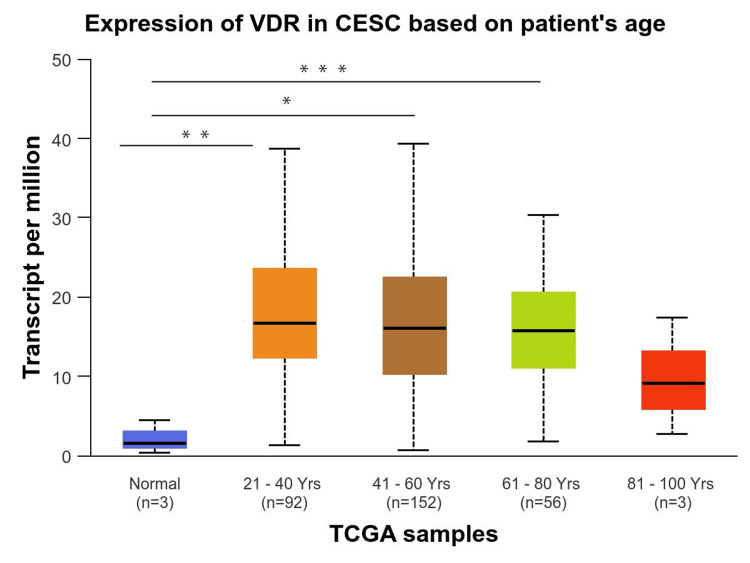
Expression of VDR in cervical squamous cell carcinoma based on patient's age Exploration of identifying the significance of VDR gene in cervical squamous cell carcinoma patients based on patient's age using the UALCAN database. VDR: 1,25-dihydroxy vitamin D3 receptor; CESC: cervical squamous cell carcinoma; num(T): the number of tumor samples; num(N): the number of normal samples.

**Figure 5:**
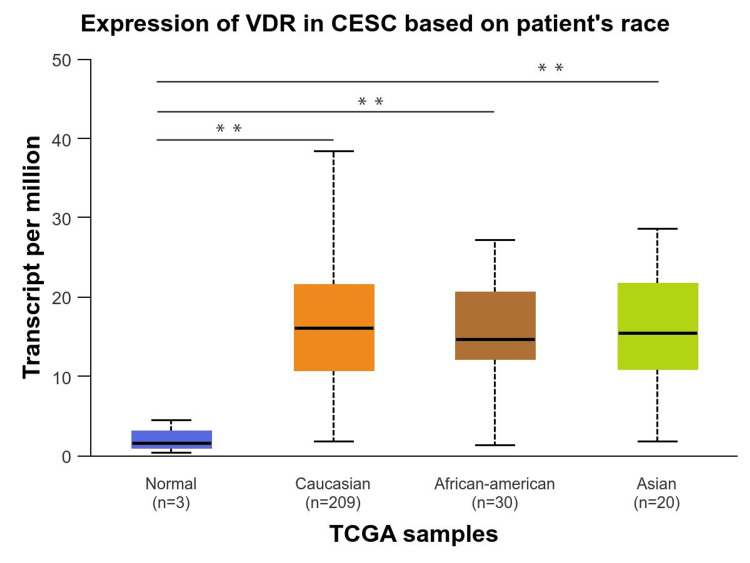
Expression of VDR in cervical squamous cell carcinoma based on patients' race Exploration of identifying the significance of VDR gene in cervical squamous cell carcinoma patients based on patient's race using the UALCAN database. VDR: 1,25-dihydroxy vitamin D3 receptor; CESC: cervical squamous cell carcinoma. num(T): the number of tumor samples, num(N): the number of normal samples.

We performed a comprehensive analysis of the relationship between VDR expression and a range of clinicopathological factors, including age, race, and cancer stage. First, we investigated how the expression of the VDR gene according to age groups was significantly upregulated in all age groups, particularly in older adults (61-80Yrs) groups with p-value (p=9.825800E-04), followed by young adult (21-40Yrs) groups and middle-aged adults (41-60Yrs). However, no significant differences were observed between the age groups (Figure 4). Based on race, VDR gene expression was significantly upregulated in all races, including Caucasians, African-Americans, and Asians compared to the normal group (p= 3.957700E-03, 4.323700E-03, and 4.216200E-03, respectively). In contrast, VDR did not show significant differences between racial populations compared to the normal population (Figure 5).

**Figure 6:**
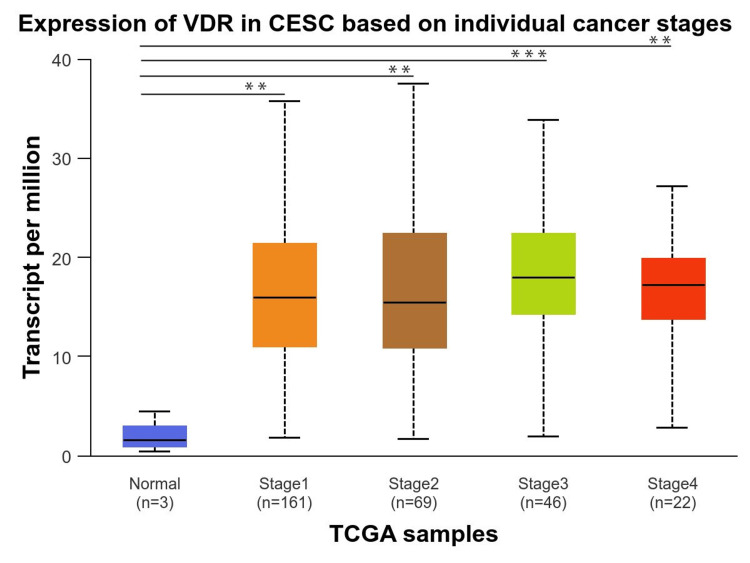
Expression of VDR in cervical squamous cell carcinoma based on individual cancer stages Exploration of identifying the significance of VDR gene in cervical squamous cell carcinoma patients based on individual cancer stages using the UALCAN database. VDR: 1,25-dihydroxy vitamin D3 receptor; CESC: cervical squamous cell carcinoma; num(T): the number of tumor samples; num(N): the number of normal samples.

Finally, we investigated the VDR expression in the individual cancer stages compared to normal individuals, which revealed that individual cancer stages 1,2,3, and 4 were significantly upregulated and the highest in stage 3 (p = 1.004560E-03), whereas VDR gene expression was not significantly different between stages (Figure 6).

Correlation of VDR with the abundance of immune cells 

**Figure 7:**
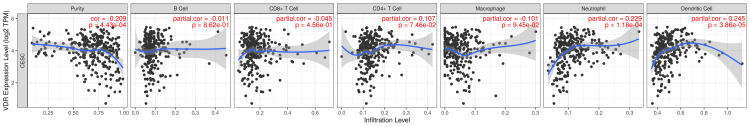
Correlation of VDR expression among CESC tumors with the abundance of immune cell filtration levels Correlation of VDR expression among CESC tumors with the abundance of immune cell filtration levels (B cells, CD8+ T cells, CD4+ T cells, macrophages, neutrophils, dendritic cells) using TIMER database cor and p value. VDR: 1,25-dihydroxy vitamin D3 receptor; TIMER: tumor immune estimation resource; CESC: Cervical squamous cell carcinoma; B cells: B lymphocytes; CD8+ T cells: cytotoxic T lymphocytes; CD4+ T cells: T helper cells. Cor: correlation; partial cor: partial correlation; p:  p value.

The expression of the VDR is associated with the presence of several immune cell types, including B cells, CD8+ T cells, CD4+ T cells, macrophages, neutrophils, and dendritic cells, as well as the purity of tumor cells in CESC. In CESC patients, VDR expression was positively correlated with neutrophils (Cor=0.229, P=1.18e-04) and dendritic cells (Cor=0.245, P=3.86e-05) and negatively correlated with tumor cell purity, but not with the abundance of B cells, CD8+ T cells, CD4+ T cells, and macrophages (Figure 7).

VDR as a prognostic biomarker in CESC

**Figure 8:**
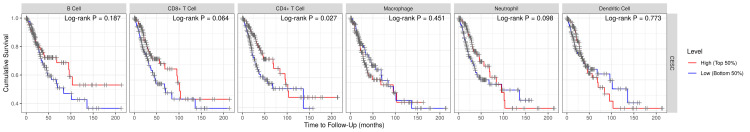
Overall survival analysis using the TIMER database The correlation between the VDR expression level and the survival outcome of patients using TIMER VDR: 1,25-dihydroxy vitamin D3 receptor; CESC: cervical squamous cell carcinoma; TIMER: tumor immune estimation resource; B cell: B lymphocyte; CD8+ T cell: cytotoxic T lymphocytes; CD4+ T cells: T helper cells; OS: overall survival; Log-rank P: p-value resulting from the log-rank test.

**Figure 9:**
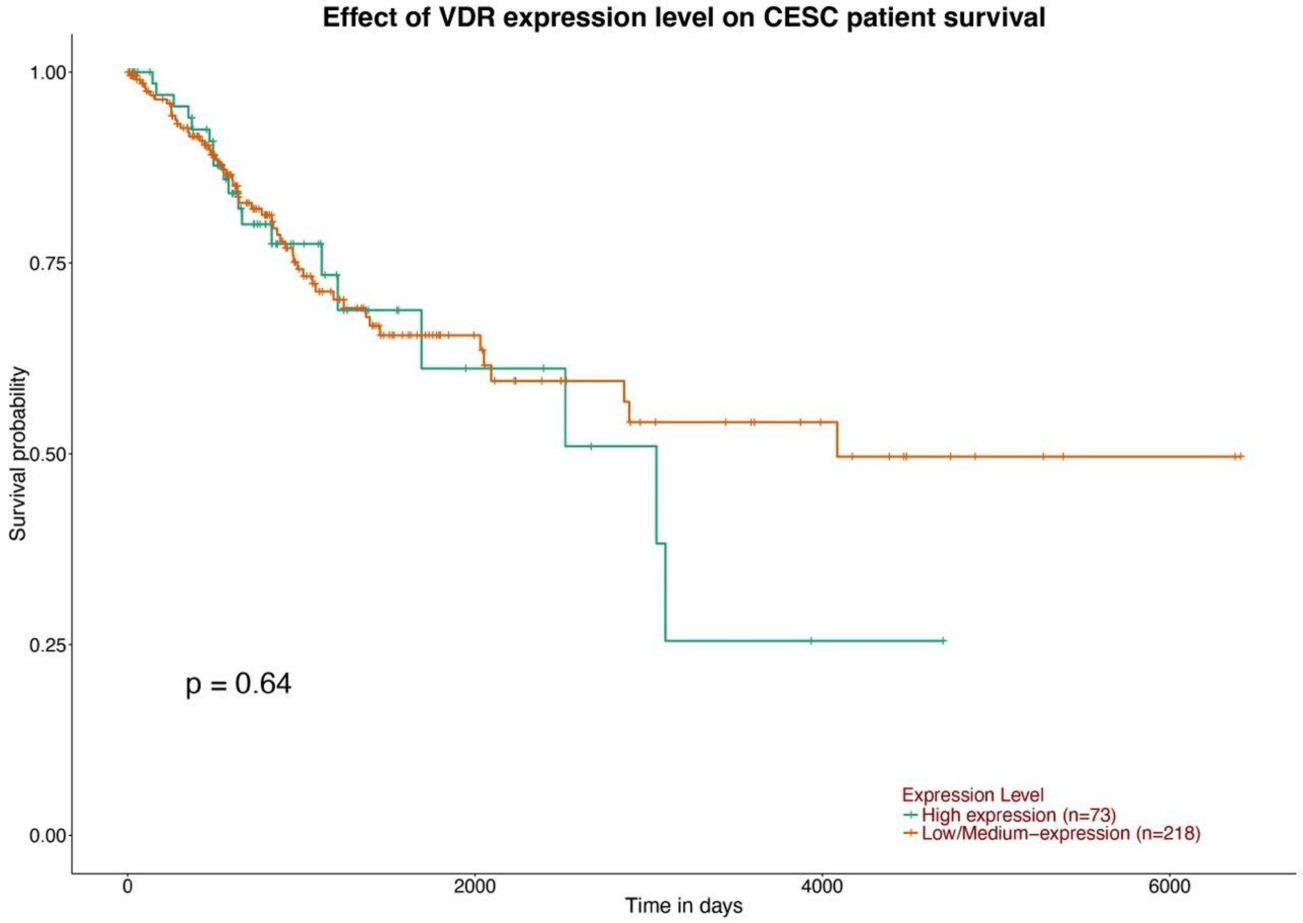
Overall survival analysis using the UALCAN database The correlation between the VDR expression level and the survival outcome of patients using UALCAN databases. VDR: 1,25-dihydroxy vitamin D3 receptor; CESC: cervical squamous cell carcinoma; B cell: B lymphocyte; CD8+ T cell: cytotoxic T lymphocytes; CD4+ T cells: T helper cells; OS: overall survival; Log-rank P: p-value resulting from the log-rank test.

Our analysis aimed to examine the association between VDR and patient prognosis in CESC, using the TIMER database. We included clinical variables such as age and immune cells in a Cox proportional hazards model to emphasize the potential impact of these factors on patient outcomes. The results of the study conducted in patients with CESC showed that VDR expression did not have a significant influence on prognosis; however, it demonstrated a positive association with CD4+ T cells (P=0.027210288) (Figures 8, 9). 

According to the Cox proportional hazards model, age and immune cells were not significantly associated with prognosis (Table 1).

**Table 1:** The Cox proportional hazard model of VDR, immune cells, and clinical parameters in CESC The Cox proportional hazard model of VDR, immune cells, and clinical parameters in CESC For 423 patients with 187 dying     *p < 0.05, ***p < 0.001. VDR: vitamin D (1,25-dihydroxy vitamin D3) receptor gene; Coef: coefficient; CESC: cervical squamous cell carcinoma; HR: hazard ratio; 95%CI_l: lower 95% confidential interval; 95%CI_u: upper 95% confidential interval; sig: significance, p: p-value.

Survival Parameters	Coef	HR	95%Cl_l	95%Cl_u	P value	Sig
Age	0.014	1.014	0.997	1.032	0.103	_
B_Cell	-2.256	0.105	0.000	940.080	0.627	_
CD8_Tcel	-4.479	0.011	0.000	1.424	0.069	_
CD4_Tcell	-6.007	0.002	0.000	6.751	0.137	_
Macrophage	2.479	11.933	0.009	15408.975	0.498	_
Neutrophil	-2.435	0.088	0.000	737.879	0.597	_
Dendritic	3.440	31.186	0.253	3845.331	0.161	_

Genetic alteration analysis of the VDR

**Figure 10:**
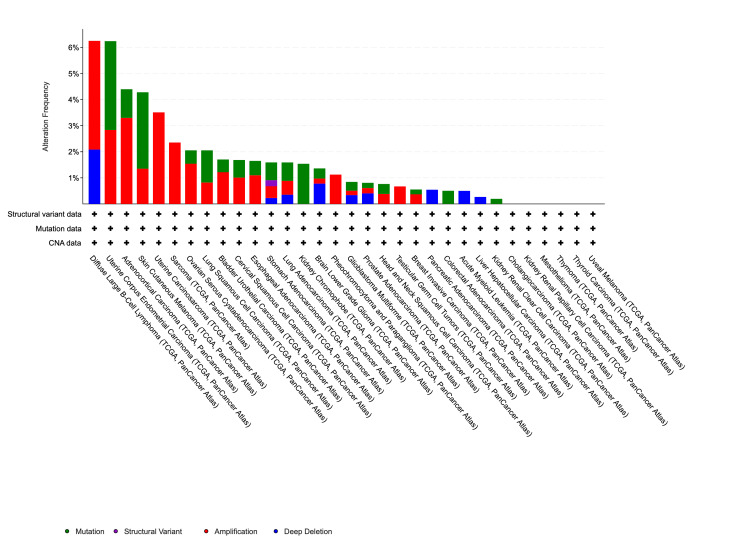
Alteration frequency of VDR in the study of various cancers In the genetic alteration analysis of VDR using the cBioPortal database, amplification mutations were the most prevalent type of VDR mutation, followed by deep deletions. VDR: 1,25-dihydroxy vitamin D3 receptor

Using the cBioportal platform, variation of the VDR across different cancers was analyzed. Our analysis of the genetic makeup of the VDR gene across various cancers was conducted using the TCGA datasets. Amplification mutations were the most common type of VDR mutations, followed by deep deletions. Also, the results indicated that the VDR gene displayed mutations in approximately 1% of the 10,967 samples from 32 queried studies. In cervical squamous cell carcinoma, we observed that the VDR gene was altered in 1.68% of 297 cases. Specifically, the amplification mutation was found in 1.01% of the three cases, while the mutation occurred in 0.67% of the two cases (Figure 10).

**Figure 11:**
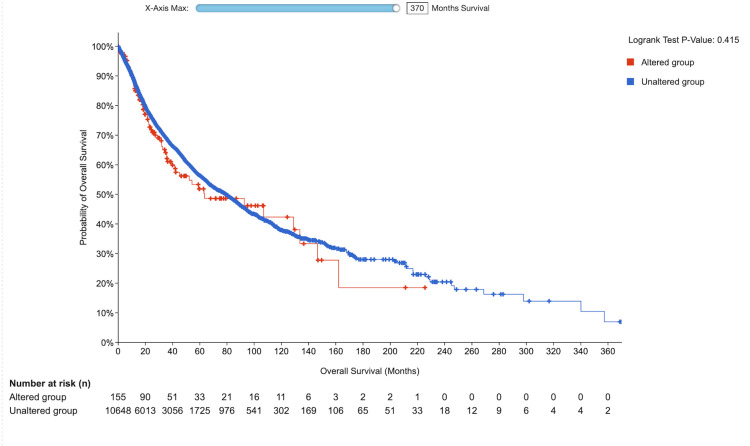
Genetic alteration analysis of VDR utilizing the cBioPortal database Correlation between survival rate of patients with different cancers and genetic alteration in VDR. CESC: cervical squamous cell carcinoma; VDR: vitamin D (1,25-dihydroxy vitamin D3) receptor gene; OS: overall survival; Log-rank P: p-value resulting from the log-rank test.

Our study revealed that a substantial proportion of the samples did not exhibit mutated VDR. Nevertheless, patients who had mutated VDR displayed a more unfavorable prognosis than those who did not possess mutated VDR (p = 0.415), (Figure 11).

Integration of public databases for cross-validation

**Figure 12:**
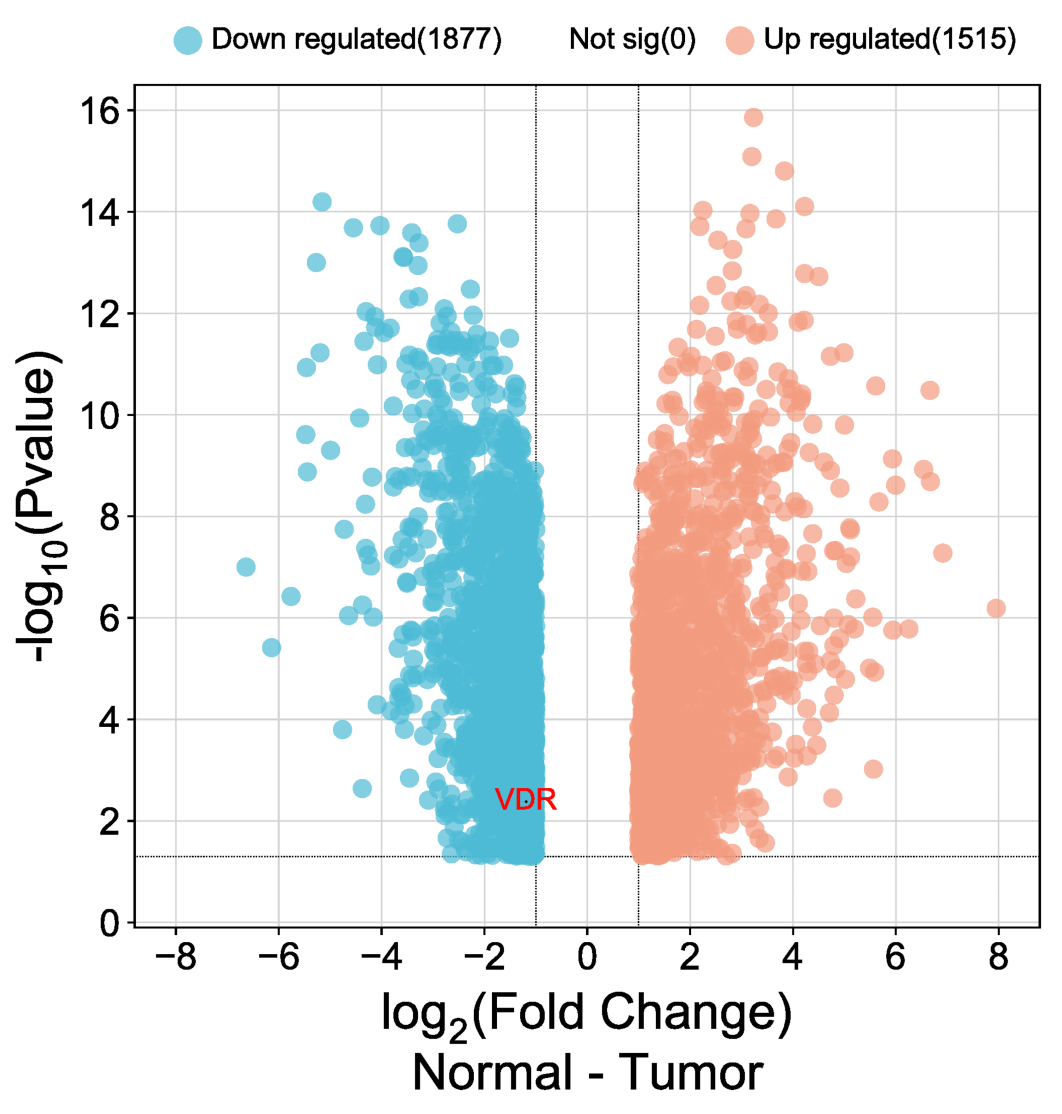
Volcano plots demonstrate the differentially expressed genes and illumination of VDR Volcano plots using the www.bioinformatic.com.cn database to highlight differential gene expression, with upregulated genes shown in orange color and downregulated genes in blue. These plots focused on gene expression changes with a logarithmic fold change >1 and an adjusted p-value below 0.05. The vitamin D receptor (VDR) was a specific focus of interest. VDR:  1,25-dihydroxy vitamin D3 receptor; CESC: cervical squamous cell carcinoma

The expression of VDR in CESC was validated using public databases from the Gene Expression Omnibus (GEO). We employed the GEO2R tool to indicate the differential expression of VDR (|Log2FC| > 1, adjusted p-value < 0.05) and used www.bioinformatic.com.cn to visualize volcano plot differences in gene expression. The database was GSE122697, including five patient samples of normal cervical epithelium and 11 cervical squamous cell carcinomas. The analysis revealed 1515 up-regulated and 1877 down-regulated genes. Figure 12 illustrates the down-regulation of CESC in patients using volcano plots.

## Discussion

The role of the VDR has been established in the progression and prognosis of various types of cancer, including CESC [14]. The current study conducted a pan-cancer analysis of VDR expression in CESC using TIMER, GEPIA, and UALCAN, and the results revealed that VDR expression was significantly upregulated in tumors across the three databases. Our analysis revealed a strong correlation between VDR expression and clinicopathological parameters. Specifically, VDR expression was significantly upregulated in CESC across all age groups, races, and cancer stages. However, the highest expression was observed in older adults and those with advanced cancer stages. There were no significant differences in VDR expression between different races or ages. These findings provide valuable insights into the molecular mechanisms underlying CESC and could have important implications for the development of targeted therapies.

In patients with CESC, the VDR expression is positively correlated with neutrophils and dendritic cells and negatively correlated with tumor cell purity. We investigated the correlation between VDR expression and the abundance of immune cells, including B cells, CD8+ T cells, CD4+ T cells, macrophages, neutrophils, and dendritic cells in different TCGA tumors using TIMER 2.0. We found a negative correlation between VDR expression and the abundance of B cells, CD8+ T cells, macrophages, and tumor purity; however, there was a positive correlation between the expression of VDR and the infiltration of CD4+ T cells, neutrophils, and dendritic cells. The positive association with these immune cells, coupled with the negative correlation with tumor cell purity, suggests that the VDR may influence the infiltration and activity of immune cells within the tumor, thereby affecting tumor growth and response to therapy.

We established the relationship between VDR expression and survival outcomes in patients with CESC using TIMER. We found that in CESC the VDR expression has no significant impact on the patient's prognosis p =0.415, which contradicts that of lung adenocarcinoma, and increased VDR expression has been linked to improved survival rates, potentially due to a reduction in proliferative activity and induction of G1 cell cycle arrest. This finding has been demonstrated in various studies, including [15, 16] and [17]. Furthermore, VDR displacement is associated with a lower histological grade in endometrial carcinoma, suggesting that it may serve as a valuable biomarker for disease prognosis and may guide targeted therapies.

The role of the VDR in shaping the tumor microenvironment and influencing immune responses in cervical squamous cell carcinoma has received significant attention [18]. Additionally, we explored VDR genetic variation across various cancers using the cBioPortal platform and discovered that amplification mutations were the most prevalent type of VDR mutations, followed by deep deletions. Our analysis demonstrated that 1.68% of the 297 patients had alterations in the VDR gene. Specifically, the amplification mutation was observed in 1.01% of 3 cases, whereas the mutation occurred in 0.67% of the two cases. Our findings indicated that a substantial proportion of the samples did not exhibit mutated VDR. However, patients with mutated VDR displayed a more unfavorable prognosis than those who did not possess mutated.

The expression of the VDR in CESC was validated using samples from 5 patients with normal cervical epithelium and 11 cases of cervical squamous cell carcinoma. The analysis revealed 1515 upregulated genes and 1877 downregulated genes, including vitamin D3 receptors. The VDR has garnered considerable attention in recent pan-cancer analyses, revealing its extensive implications across a variety of cancer types. These findings emphasize the importance of further exploring VDR's role as a prognostic marker and potential therapeutic target in CESC. Additionally, ongoing research continues to investigate the potential use of vitamin D analogs as adjuvant therapies for cancer. VDR has emerged as a critical element in cancer biology, and its expression and function have been linked to various cancers. 

Several studies have highlighted the potential of VDR as a prognostic and therapeutic target for CESC, a prevalent gynecological cancer [19-21].

This study provides valuable insights into the function of the VDR in CESC, shedding light on the intricate relationship between vitamin D signaling, tumor biology, and the immune system. Ultimately, this knowledge may be used to develop innovative therapeutic strategies and personalized treatment approaches for patients with this aggressive type of cancer. Several studies have shown that patients with higher levels of VDR have better survival rates than those with lower levels, emphasizing the predictive significance of VDR expression in CESC. We found that VDR expression was significantly downregulated in CESC compared to normal tissues, which could encourage the potential use of VDR as a prognostic biomarker in this cancer.

Study limitations

The prognostic and immunological implications of the VDR in CESC through pan-cancer analysis are valuable but have limitations. The individual patient differences, including genetic background and treatment history, can affect VDR's role of the VDR in prognosis and immunology. The methodological and technical limitations, such as the accuracy and reliability of the findings depend on the bioinformatic tools and algorithms used, which can lead to varying interpretations. The lack of functional studies means that pan-cancer analysis provides correlative data, necessitating functional studies to establish causality and to understand the mechanistic role of VDR in CESC. Overcoming these limitations requires a multidisciplinary approach combining advanced bioinformatics, robust experimental validation, and clinical studies to fully understand VDR's role of VDR in CESC and its potential as a prognostic and immunological marker.

## Conclusions

The outcomes of pan-cancer analysis have highlighted the prognostic and immunological importance of the VDR in cervical squamous cell carcinoma; and indicated that VDR expression was positively correlated with neutrophils and dendritic cells and negatively correlated with tumor cell purity in patients with CESC. There was no significant correlation between VDR expression and the abundance of B cells, CD8+ T cells, CD4+ T cells, and macrophages.

Our study found no significant effect of VDR expression on patient prognosis, although it positively correlated with CD4+ T cells. The Cox proportional hazards model indicated that age and immune cells did not significantly affect prognosis. These findings indicate a connection between elevated levels of VDR expression and enhanced survival rates, as well as the existence of an unfavorable immune microenvironment. Therefore, VDR may be a promising biomarker.
